# Dr. J. Marion Sims

**Published:** 1875-08

**Authors:** 


THE NEWLY ELECTED PRESIDENT.—J. Marion Sims, M.D., the newly elected president of the National Medical Association, is a native of South Carolina. He was born January 25, 1813, and is, of course, sixty-two years of age, though he has the appearance of a much younger man. He graduated at Jefferson College, in Philadelphia, in 1835, and settled in Montgomery, Ala., where he practiced with great success for eighteen years, when failing health induced him to remove to New York. There he founded that eminent charity, the Woman's Hospital, and advanced with rapid strides toward the exalted position he now occupies in the specialty of gynæcology. When the War of the Rebellion broke out, his sympathies running strongly in favor of his native South, he left his country and took up his abode in Paris. A most remarkable career of distinction in Europe now opened upon him. Honors of the highest character were conferred on him in France, Belgium, Germany, Spain, Portugal and Italy, and in England. In 1868 he returned to America, and has since resided in New York. He has a son engaged in the practice of medicine, and several other children. Probably no other man in the world holds a higher position than he occupies in the medical profession, nor has any other one been the recipient of regal honors from so many sources.—*Pacific Medical and Surgical Journal.*

